# A Concise and Systematic Review on Non-Invasive Glucose Monitoring for Potential Diabetes Management

**DOI:** 10.3390/bios12110965

**Published:** 2022-11-03

**Authors:** Soumyasanta Laha, Aditi Rajput, Suvra S. Laha, Rohan Jadhav

**Affiliations:** 1 Department of Electrical and Computer Engineering, California State University, Fresno, Fresno, CA 93740, USA; 2 Centre for Nano Science and Engineering (CeNSE), Indian Institute of Science, Bangalore 560012, India; 3 Department of Public Health, California State University, Fresno, Fresno, CA 93740, USA

**Keywords:** optical spectroscopy, photoacoustic spectroscopy, electromagnetic sensing, nanomaterials, diabetes management

## Abstract

The current standard of diabetes management depends upon the invasive blood pricking techniques. In recent times, the availability of minimally invasive continuous glucose monitoring devices have made some improvements in the life of diabetic patients however it has its own limitations which include painful insertion, excessive cost, discomfort and an active risk due to the presence of a foreign body under the skin. Due to all these factors, the non-invasive glucose monitoring has remain a subject of research for the last two decades and multiple techniques of non-invasive glucose monitoring have been proposed. These proposed techniques have the potential to be evolved into a wearable device for non-invasive diabetes management. This paper reviews research advances and major challenges of such techniques or methods in recent years and broadly classifies them into four types based on their detection principles. These four methods are: optical spectroscopy, photoacoustic spectroscopy, electromagnetic sensing and nanomaterial based sensing. The paper primarily focuses on the evolution of non-invasive technology from bench-top equipment to smart wearable devices for personalized non-invasive continuous glucose monitoring in these four methods. With the rapid evolve of wearable technology, all these four methods of non-invasive blood glucose monitoring independently or in combination of two or more have the potential to become a reality in the near future for efficient, affordable, accurate and pain-free diabetes management.

## 1. Introduction

Diabetes mellitus ranks among the top ten lethal disease globally as approximately 451 million people suffer from diabetes across the world according to WHO estimates [1]. As of 2020, in the US alone, the prevalence of diabetes is officially estimated to be around 37.3 million [2], approximately 11% of the total US population. The actual number could be much higher because of unavailability of adequate/efficient infrastructure and/or lack of willingness to preventive check-ups among the population.

The non invasive blood glucose monitoring has the potential to become wearable and continuous in the near future is particularly important for patients with hypoglycemia which is known to cause death during sleep due to unexpected changes in blood glucose levels and is a common occurrence among insulin-dependent diabetics [3]. Hypoglycemia unawareness results from reduced sympathetic adrenal response and patients have difficulty to recognize hypoglycemia events which puts them at high risk of adverse health events [3].These adverse health events include cardiovascular ischemia [4], dementia [5], falls [6], and even deaths [7]. Hypoglycemia-related events resulted in 100,000 visits to emergency rooms and 30,000 hospital admissions between 2007 and 2011 in the US [8]. On the other hand, poorly managed hyperglycemia can also lead to adverse health events such as cerebrovascular accidents, cardiovascular events, and peripheral vascular disease [9]. Diabetic ketoacidosis (DK) and Hyperglycemic hyperosmolar state (HHS) are life-threatening emergencies among diabetic patients. Patients with oliguria, unconsciousness for a period tend to have poor prognosis. Complications like cerebral edema, hypokalemia, rhabdomyolysis (more common during HHS) and respiratory failure due to secondary infection can occur [10]. Blood sugar reaches more than 250 mg/dL in DK and 600 plus in HHS [11]. Thus, prompt and real time identification of rapid fluctuations in the blood sugar is the key to successful management of diabetes-related emergencies and increases the likelihood of better prognosis. Thus, noninvasive blood glucose monitoring which has the potential for real time diagnosis can be used to identify the development of DK and HHS early and avoid all the aforementioned complications without any pain.

As a result of these, there have been multiple research articles published on various types and techniques of noninvasive glucose monitoring in the last two decades. Multiple review articles have also been published highlighting and discussing the progresses and challenges of these original research. The current review paper *distinguishes* from the previously published review articles by discussing recent papers in a concise manner using four detection methods widely investigated for noninvasive glucose monitoring. In this paper, the classification of these four methods are based on the detection principles as explained in Section 3. Furthermore, the *uniqueness* of the paper comes from the systematic progress in reporting original research papers on noninvasive glucose monitoring starting with tabletop *in-vitro* sensing to *in-vivo* wearable/compact/portable continuous blood glucose monitoring with advanced technology (such as Machine learning/Internet of Things (IoT)/advanced nanomaterials etc.) or its high possibility in near future. To exemplify, the classification of sensing in the current paper is based on the principles of excitation and detection of glucose unlike [12] that is based on the location where the sensor is placed or on the types of human excretion (saliva, tears, urine etc.). In [13], developments of seven optical noninvasive glucose monitoring techniques are reviewed without reporting on the electromagnetic and nanomaterial based sensing, whereas in [14], the use of microwave planar resonant sensors to track changes in glucose concentration has been only assessed. In [13,15], photoacoustic spectroscopy has been classified under optical methods unlike the current paper where it has been differentiated from the optical approach based on its detection principle of acoustics. Recent developments on non-invasive machine learning and neural network methods are discussed in [16]. None of these papers focus on the technology evolution from bench-top to a wearable device.

## 2. Background

This section describes the two currently used modes of blood glucose monitoring for diabetes management. They are the conventional invasive blood extraction and pricking technique and the recently popular minimally invasive continuous glucose monitoring.

### 2.1. Diabetes Management via Conventional Blood Pricking Invasive Device

The gold standard for the determination of blood glucose concentration is the Hexokinase Method [17]. This procedure involves the extraction of a 1.5 mL blood sample using a specialised laboratory equipment. The method is not portable, real time and cannot be operated in home environment. Due to this, a method that relies on using an invasive finger pricking approach has become the most widely used standard of successful management and effective treatment of diabetes. This makes use of capillary glucometers, where a test strip interacts with with a drop of blood, extracted by the pricking in the finger [18]. However, the finger prick technique is not necessarily accurate [19].

Some techniques have evolved to make the these sensors to be more accurate. To make insulin prediction with less amount of invasive procedure and some degree of accuracy, a local fuzzy reconstruction method based on chaos theory for predicting fasting blood glucose at peak time was reported in [20]. The prediction achieves a 70–90% success rate. In recent times, techniques based on machine learning has been introduced for insulin prediction [21] for similar reasons.

However, these techniques do not eliminate the greatest agony of the invasive procedure: pain and discomfort. In addition, there is always a high probability for the finger pricking procedure to cause infection. The daily usage of lancets and testing strips causes potentially unsafe bio-hazards. Furthermore, a pre-emptive action on the part of the patient or caretaker is always necessary for the drawing and testing of blood. As earlier explained, for patients with complex medical conditions and aggressive forms of type-2 diabetes, continuous monitoring is highly desired so that unexpected changes in blood glucose levels, known to cause death due to sudden hypoglycemia during sleep, can be detected.

### 2.2. Diabetes Management via Minimally Invasive Continuous Glucose Monitoring

The extreme fluctuations in the blood sugar level can cause cardiovascular events [4,22] and even deaths. Therefore, there is a need to monitor blood glucose levels continuously [23]. Continuous glucose monitoring (CGM) devices can be helpful to identify these extreme fluctuations in the blood sugar and can alert the patient thereby enhancing self or parental management which can lead to avoidance of severe outcomes related to hypo or hyperglycemia. Very recently, multiple such commercial (CGM) devices (Dexcom [24], Adobe FreeStyleLibre [25], Eversense [26] etc.) have come up but none of them is completely non-invasive and wearable as it needs some part to be periodically implanted subcutaneously. Current versions of CGM uses a metal inserted into the skin and remains in contact with blood. It monitors blood sugar level by measuring the charge on the electrical current which emerges from the oxidation of glucose from the enzymes that the device releases. Besides the painful insertion, discomfort and the risk due to the presence of a foreign body under the skin always remains active. Moreover, the adhesives used in CGM systems can cause skin irritation and contact dermatitis in some cases [27]. These skin problems can cause emotional distress among diabetics and can inversely affect adherence to CGM [28]. Current versions of CGM are semi-invasive where a metal is inserted into the skin and serves as a sensor. It emits blood sugar oxidizing enzyme. The interaction of this enzyme with blood sugar molecules results in the formation of hydrogen peroxides amongst other compounds. The reaction also results in the generation of a current. The charge on this current is measured and it corresponds to the appropriate amount of blood sugar in the interstitial cells. The lag time currently is 1 to 5 min [23]. Although the CGM systems have been quite accurate and efficient in monitoring blood glucose levels among insulin-dependent diabetics [29], high costs particularly among those who are uninsured or underinsured and difficulty in placement of these devices on the body and the dislike of skin attachment of the subcutaneous implant were among the major barriers, particularly among adolescents [30,31]. Therefore, there is a need of a strategy that is non-invasive, affordable but accurate at the same time at par with CGM and traditional invasive methods. To this end, numerous research findings thus far suggest that a workable complete non-invasive blood glucose monitoring solution is feasible and multiple techniques are being pursued to build a glucose monitor that is non-invasive, accurate, wearable, low-cost and continuous.

## 3. Principles of Non Invasive Glucose Monitoring

Multitude principles and techniques of non-invasive glucose monitoring have been pursued in academic research as well as in industry in recent times. Among them, broadly four particular non-invasing glucose monitoring principles are widely investigated and reported. These four principles are differentiated based on the their principles of detection of glucose. They are as follows:

- Optical Spectroscopy (Optical Detection)
- Photoacoustic Spectroscopy (Acoustic Detection)
- Electromagnetic Sensing (Electromagnetic Detection)
- Nanomaterial Based Sensing (Electrochemical Detection)

**Figure 1:**
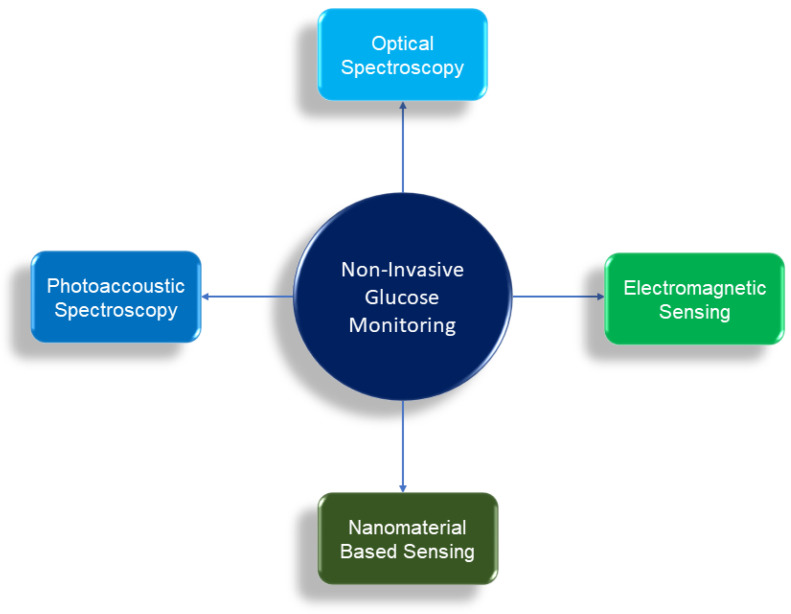
The four different types of noninvasive glucose monitoring technique are illustrated based on their principle of detection.

These four techniques are illustrated in Figure 1 and are reviewed in Section 4, Section 5, Section 6 and Section 7. The principles of these four techniques are explained in the following subsections.

### 3.1. Optical Spectroscopy

The detection principle of the Optical Spectroscopy is based on the fact that glucose influences the optical signal passing through it by absorption of light at some particular overtones and combination band wavelengths in the mid infrared (mid-IR) and near infrared (NIR) spectrum regions. Among the mid-IR spectrum of glucose light interactions, a first OH overtone band is designated at 1408 nm. The 1536 nm band can be designated as an OH and CH combination band whereas, the 1688 nm band is designated as a CH overtone band [32]. In the NIR spectrum, the combination bands are a second OH overtone band at 939 nm and a second harmonic CH overtone band at 1126 nm [32]. Due to this absorbance at these particular wavelengths, the intensity of light transmitted through the glucose either *in-vitro* or *in-vivo* decreases as concentration of the glucose increases and vice versa. Using optical sensors like photodiodes, this phenomenon is utilized in the measurement of the blood glucose concentration from the transmitted or reflected intensity of light [32]. Another way to measure glucose concentration using optical detection is Surface Plasmon Resonance (SPR), where a beam of light is passed through the prism on the back of the SPR metal surface which then bends on to the detector. Unlike the intensity of the light, the method exploits information on angular shift to identify the glucose concentration. At a certain resonance angle (refractive index), the light excites the electrons on the metal part of the chip. When an analyte is introduced to the SPR surface, the shift of the reflection intensity curve is observed. The direct measurement of blood sugar is based on the change in angle of the SPR reflection intensity curve [33].

### 3.2. Photoacoustic Spectroscopy

The photoacoustic glucose monitoring is a hybrid approach, which combines optical excitation and acoustic detection in determining the glucose concentration. Here, the optical energy from the excitation is converted into an acoustic energy by a multistage energy conversion process [34]. The optical excitation of the glucose molecules in blood leads to the localized heating of the solution, which produces a small temperature rise, resulting in the volumetric thermal expansion of the optical interaction region. The associated ultrasonic pressure pulse is then measured by an ultrasonic piezoelectric transducer, which is then converted into electrical signals for determining the glucose concentration.

### 3.3. Electromagnetic Sensing

The frequency dependency of body tissue has been investigated since early 20th century. The propagation of electromagnetic waves through any media depends on the permittivity of that media. The permittivity is a frequency-dependent parameter unique for each material. These investigations have found that the complex permittivity of tissue is strongly frequency dependent. The underlying mathematical principle is derived from the Debye’s relaxation theory. The mathematical equations can be written as [35,36]:

(1) $$\epsilon_{r}\left(\omega ,\chi \right)=\epsilon_{\infty }\left(\chi \right)+\frac{\epsilon_{stat}\left(\chi \right)-\epsilon_{\infty }\left(\chi \right)}{1+j\omega \tau (\chi )}$$

where, $\epsilon_{r}$ (ω) is the complex and frequency-dependent relative permittivity of a dispersive material, $\epsilon_{stat}$ is the static permittivity at lower frequencies, $\epsilon_{\infty }$ is the permittivity at high frequencies and τ is the characteristic relaxation time of the medium. ω and χ are the angular frequency and the concentration, respectively.

Human blood consists of 55% of plasma. The blood plasma consists of around 90% of water. Water being a dipolar compound, the polarization is high. This makes the relative permittivity to be high as well. In contrast, glucose has a smaller relative permittivity, being less polarized. Thus, the overall increase or decrease in glucose concentration in the same volume of blood sample reduces or elevates the relative permittivity of blood plasma, respectively. The measure of the relative permittivity by multiple techniques of electromagnetic sensing at a particular microwave/mm-wave frequency gives the value of the glucose concentration in blood in the form of measured electrical power in a very effective manner which helps in the measurement of blood glucose. In other words, the common measurement principle of these sensors is the electromagnetic interaction between the sensor and the material under test (MUT) (either blood or glucose solutions in cuvette) where the amplitude and/or phase variations in the scattering parameters are measured, when the dielectric properties of the MUT change. It is important to note that the dielectric properties of blood are modified because the change in blood glucose levels is of much greater extent than due to changes of other compounds present in the blood [37,38].

### 3.4. Nanomaterial Based Sensing

The emergence of nanomaterials, starting from metallic gold, silver, copper oxide, iron oxide to polymer composites, carbon nanotubes and graphene, as primary components in sensing technologies, has significantly upgraded the modern-day biosensors, extracting valuable physiological data and useful information from essential body fluids like urine, saliva, sweat and tears. The impact of nanomaterials in sensing applications is remarkable as they exhibit large surface area, enhanced sensitivity and selectivity, improved catalytic activities which are essential prerequisites for an accurate and precise estimation of glucose levels in humans [39,40].

## 4. Optical Spectroscopy

The optical spectroscopy is perhaps the most popular among all the four methods for non-invasive blood glucose monitoring. As explained earlier, glucose responses to light at specific wavelengths in the near infra red (NIR) and mid-infra red (mid-IR) regions. The Light Emitting Diode (LED) is mostly used for the excitation in the NIR region with some exceptions [41], however, the use of laser is seen in all cases of mid-IR regions. The signal from either the LED or the laser is pulsed at a certain frequency and is allowed to fall and pass through the glucose. One of the wavelengths at the NIR region is at 940 nm and multiple experiments have been reported at this wavelength using the transmittance principle of glucose light from using a simple operational amplifier [42] to digital detection and processing [41,43]. In the method transmittance spectroscopy, the excited optical light is allowed to transmit through the layers of epidermis, dermis and subcutaneous tissue of the finger tip, before it is detected by a photodetector.

**Figure 2:**
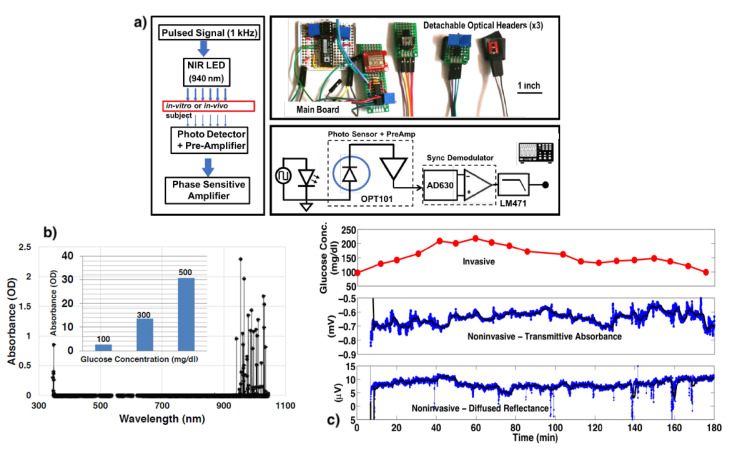
(**a**) Experimental set up of NIR spectroscopy with LED (**b**) *In-vitro* NIR spectra of glucose in water solution. Light absorption is proportional to glucose concentration (Inset). (**c**) *In-vivo* results from experiments on a human subject. Comparison of the transmittance and diffused reflectance techniques with the blood-pricking invasive technique following OGTT protocol. Reproduced with permission from [49]. Copyright 2018, IEEE.

In addition to the transmittance another method was proposed using the dermis tissue spectra [44]. Here the glucose content in dermis tissue traces the variations in blood glucose and the epidermis acts as an interference in skin tissue. This method is termed diffused reflectance. Multiple other works that have used this technique to determine the blood glucose solution are reported in [45,46,47,48]. In [49], a phase sensitive front end based compact design has been reported for continuous wearable applications. The protocols of Oral Glucose Tolerant Test (OGTT) was used to compare with the invasive blood pricking technique. This work uses both the transmittance and the diffused reflectance approach. This work has been depicted in Figure 2 as an example of optical spectroscopy with both transmittance and diffused reflectance techniques.

Cardoso et al. [50] implemented an architecture composed of two light emitters, with a wavelength of 940 nm and 805 nm. Another work [51] used 950 nm in addition to 940 nm and have shown that the output voltages are nearly linear with the increment of glucose concentration. In [52], average value of three different wavelengths was used to determine the blood glucose concentration. Besides the popular 940 nm, two other wavelengths at 850 nm and 880 nm were used. Comparative analysis with an invasive technique following either OGTT protocol or Clarke Error Grid gives a good accuracy for the non-invasive device.

Analysis with classification based machine learning models was carried out in [53]. In this work, in addition to 940 nm, transmission measurements show a high correlation value of 0.98 between glucose concentration and transmission intensity for three other wavelengths: 485 nm, 645 nm and 860 nm. The feasibility of a cellular automata based tracking method of blood glucose through skin impedance measurement was introduced in [54]. Recently, Yan et al. [55] using a portable NIR Spectrometer and advanced machine learning models have shown the model based on the combination of synergy interval, genetic algorithm and extreme learning machine as the most accurate for blood glucose detection. A partial least squares method was used in [56] to obtain a correlation coefficient of 93.2% and a prediction error of 0.23 mmol/L.

Mid-infra red spectroscopy is another mode within the optical spectroscopy used to determine glucose concentration. The glucose has a better response in some selected mid infrared bands than the NIR bands as reported in [32]. Using a laser diode, the prospect of mid-infrared spectroscopy was reported in [57] for concentrations between 75 and 160 mg/dL. Partial least squares regression was employed to predict the glucose concentration. An average error of 2% in comparison to a commercial electrochemical sensor was observed. In another study [58], the mid-infrared spectroscopy is used in the range of 3333∼2857 nm and a strong correlation between the transmittance approach and blood glucose concentration was presented. Using Fourier transform infrared spectroscopy (FT-IR) of attenuated total reflection, the spectra of a human finger was measured. Further, in [59,60,61], the techniques of Fourier transform infrared spectroscopy (FT-IR) of attenuated total reflection (ATR) were used to accurately determine the glucose concentration.

A customised multi-modal spectroscopy combining impedance spectroscopy and multi-wavelength infrared spectroscopy is proposed in [62] as an application specific integrated circuit (ASIC) in 0.18 μm 1P6M CMOS technology. It occupies an area of 12.5 mm${}^{2}$ and dissipates a peak power of 38 mW. The three wavelengths used are 850 nm, 950 nm and 1300 nm. The work further uses an artificial neural network (ANN) algorithm to achieve high accuracy in glucose estimation value. The use of ASIC together with ANN enabled the sensor to be area and power efficient for practical wearable applications of blood glucose monitoring with high accuracy. Together with the application of Healthcare IoT [63], the potential of this approach for diabetes management seems to be very bright.

In the technique of SPR, the researchers used attached glucose/galactase binding protein to SPR surface and detected blood glucose and determined the binding equilibrium of glucose molecules to the SPR surface [64]. The analysis of this equilibrium can be a key in continuous glucose monitoring. The SPR method can yield a high sensitivity in the detection of blood glucose at 1050 nm/refractive index unit [33] The sensitivity was reported to be highest at the refractive index of 0.982 nm [65]. Another study reported similar result with greatest sensitivity with refractive index in the range of 1.0000 to 1.0007 [66]. In [67], imprinted hybrid microgels achieved root-mean-squared error of predication (RMSEC) as low as ~0.2 mg/dL over a clinically relevant glucose concentration range of 1.8–360 mg/dL. In case of SPR techniques and other similar techniques that resort to large bench-top equipment for measurement and analysis, the prospect of down-scaling the technique in form-factor to that of a wearable device is minimal. Those techniques such as Optical Polarimetry, Raman Spectroscopy, Optical Coherence Tomography are not discussed in this review and can be referred in [13,15].

The accuracy of optical spectroscopy is affected by several factors which include, skin color and temperature and therefore an algorithmic correction is required [15]. According to some studies, skin can cause birefringence of light due to scattering of contents in the skin leading to lesser degree of polarization of light than expected [15,68]. The detection requires person specific calibration to mitigate the skin color and advanced machine learning algorithm to separate signals coming out of proteins and lipids [69].

## 5. Photoacoustic Spectroscopy

A large amount of work on glucose monitoring with photoacoustic spectroscopy have been also reported. As earlier explained, although this technique shares the same excitation principle with the optical spectroscopy however the detection mechanism is entirely different and is based on the principles of acoustics. The feasibility of photoacoustic spectroscopy with the depth profiling of a skin model for non-invasive glucose measurement was reported in [70]. The measurable depth of 2–3 mm with a modulation frequency of 1000–2000 Hz provided the necessary confidence to work with this technique. This led to an early work that investigated the feasibility of photoacoustic spectroscopy as an independent technique for blood glucose monitoring and reported in [71]. This work implemented a laser diode to excite the glucose medium and the signal was detected using a piezoelectric transducer followed by a low noise amplifier. The validation was implemented using the established OGTT method. It is important to note that the laser excitation in the cases described above, were pulsed at a certain frequency and is allowed to fall and pass through the glucose.

**Figure 3:**
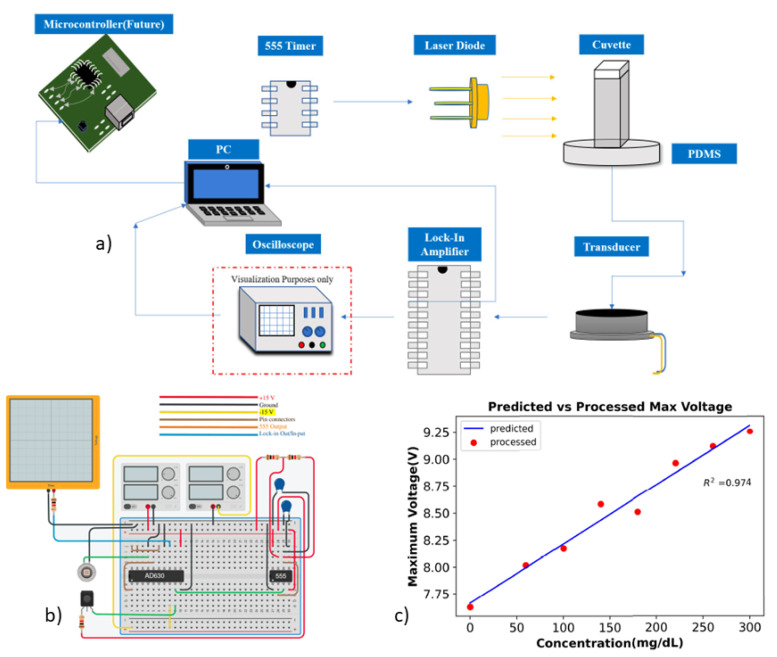
(**a**) Compact instrumentation for in vitro glucose measurement with phase sensitive detection using PAS. All the instruments are portable and can be replicated for wearable in vivo glucose monitoring (**b**) The electronics circuit for the optical stimulation and the phase sensitive detection of the proposed compact instrumentation system using the micrchip AD630 (**c**) Linear regression calculations of the Amplitude of the signal. Reproduced with permission [74]. Copyright 2022 IEEE under CC BY.

Optical excitation, where laser was not pulsed but continuous were also reported. Such continuous laser excitation with desktop based lock-in-amplifiers were reported for accurate phase-sensitive detections in [72] and later differential continuous-wave photoacoustic spectroscopy (DCW-PAS) in [73]. The DCW-PAS technique utilizes amplitude modulation of dual wavelengths of light to determine changes in glucose concentration. Both of these works also use the laser diode for the optical excitation. Very recently, Faheem et al., has implemented a laser based photoacoustic glucose monitoring system using a phase sensitive amplifier in wearable form factor as reported in [74]. This work replaces the desktop based lock-in amplifier with an integrated modulator/demodulator microchip working as a lock-in-amplifier for practical wearable applications in diabetes management. The work further analyzes the results using classification based machine learning algorithms and achieve a correlation coefficient of 97% and p-value 5.6e-6. This work has been depicted in Figure 3 as an example of photoacoustic spectroscopy with compact instruments for potential wearable applications. In [75], a portable photoacoustic embedded system is implemented using field programmable gate array (FPGA). The glucose measurement technique is verified both *in-vitro* and *in-vivo*. This work further advances to include a kernel-based regression algorithm using multiple features of the photoacoustic signal to estimate glucose concentration [76]. The work also uses cloud service using a mobile device in as an example of Healthcare IoT for glucose monitoring. Some other works that were reported and used laser diodes are [77,78,79,80].

In comparison to the optical spectroscopy, this technique is yet to gain similar popularity. This is because the optical excitation in this technique depends mostly on laser, unlike an LED as in the case of the NIR Optical Spectroscopy. The laser has multiple safety concerns and health issues and is therefore not recommended for continuous blood glucose monitoring. A major challenge to replace it with NIR/mid-IR LED that has the potential to be a continuous wearable system will be to compromise on the signal to noise ratio (SNR) of the detected signal. In this method, the SNR is comparatively less than the optical approach because the acoustic signal coming as an electric signal out of a transducer is relatively weak in comparison to a direct optical detection with a photodetector as in the case of an optical approach. A few works have attempted to mitigate this issue. In [81], an NIR LED based photoacoustic glucose monitoring system is reported. They make use of a box that has a good sound shielding characteristic to attenuate noise from outside without compromising on the SNR. The challenge to improve the SNR quality can be further resolved by implementing different signal processing techniques such as signal averaging reported in [82,83] in the context of photoacoustic imaging.

The measurement of blood glucose using this method is also challenged by skin thickness, roughness and moisture and other skin conditions [69]. These factors interfere the absorption of light in the skin and the glucose signal will not be as strong. The presence of other contents in the blood such as lipids and proteins. Similar to the optical approach, person specific calibration, signal averaging and advanced machine learning techniques will be able to mitigate these effects.

## 6. Electromagnetic Sensing

The electromagnetic sensing can be further categorized broadly into Planar Microwave Resonant Sensors and Antenna Sensing, based on the instrument used and principles of microwave theory. They are described as follows:

### 6.1. Planar Microwave Resonant Sensors

Planar microwave sensors are considered an attractive choice to non-invasively probe the dielectric attributes of biological tissues due to their low cost, simple fabrication, miniature scale, and minimum risk to human health. This method relies on the change in the permittivity of blood due to the variation in glucose content resulting out of small shifts in the sensor’s frequency response. However, there are certain challenges that should be taken into consideration. In [84], Volkan et al., has investigated the external factors those play a major role in the resonant sensor’s response, making it challenging to achieve the accuracy needed for blood glucose measurements in these small shifts in frequency.

**Figure 4:**
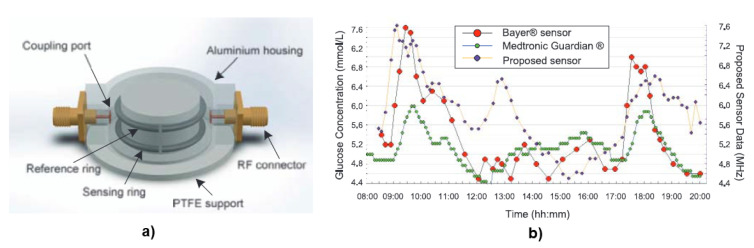
(**a**) A 3D structure of a double split-ring resonator sensor. (**b**) Noninvasive sensor data against the commercially available continuous invasive sensor (Medtronic) and blood strip glucometer (Bayer). Reproduced with permission [85]. Copyright 2014 IEEE.

A split-ring resonator has been used in [85], to measure the glucose concentration at 1.4 GHz. The sensor has two spatially separated rings, one of which interacts with the change in glucose level of the device under test while the second ring is used as a reference. The observed OGTT trend showed nice correlation of frequency shift and the glucose concentration comparable to a commercial sensor, including a CGM device and a blood pricking device. This work has been depicted in Figure 4 as an example of planar microwave sensors. In [86], the sensor has a microfluidic container on top and works at a frequency of 2 GHz *in-vitro*. The sensor responds to permittivity change with glucose solutions at varying concentrations. The range of glucose concentrations used are from 50 mg/dL to 300 mg/dL. A multi-layer is approach is proposed in [87]. The paper analyses three different microwave resonator structure and finds the ground plane coplanar waveguide to be the most suitable amongst them. In [88], a microwave resonator comparable to the size of a coin has been fabricated. The device was sensitive enough to detect as low as 10 mg/dL change in glucose concentration.

In [89], a metamaterial structure monitors the variation of blood glucose concentration on human body and increases the sensitivity by the addition of a diode. The change in frequency and magnitude (reflection loss) of 1.4 MHz and 0.7 dB, respectively, increases to 5.2 MHz and 1.2 dB, respectively. The improvements exploit the nonlinear effects of the diode. Another microwave sensor is suggested with a sensitivity performance of about 6.2 dB/(mg/mL) that operates in the range of 1–6 GHz [90]. The sensor gets excited by a coupled microstrip transmission-line that is etched on the bottom side of the substrate and monitor the glucose concentration changes by recording the frequency response of the maximum reflection and transmission loss. An interdigital capacitor sensor was reported in [91] at 4.080 GHz. The measurement principle here is based on the fringing electric field produced on top of the interdigital capacitor sensor of the MUT. In [92], an ultrawideband microwave technique has been proposed for non-invasive blood glucose monitoring in the frequency range of 3–10 GHz. In this frequency range, there is a regularity of the energy of the received signal in the blood glucose concentration of varied from 70 to 400 mg/dL. The technique uses a single pair of antennas to achieve this. The gain ranges from 0 to 2 dB for blood glucose level from 50 to 400 mg/dL.

The transmission properties of glucose solutions with different concentrations, including the amplitude and phase responses, are reported in [93]. With modeling and experiment, two glucose solutions utilizing saline and distilled water are studied at 17 GHz Ku-band. At 40 GHz, a sensitivity of 0.2${}^{\circ }$ phase shift per 10 mg/dL was achieved in [94]. The transmission phase of glucose solutions is shown to have a linear response to glucose concentration from hypoglycemia to hyperglycemia, indicating that microwave technology in the Ku-band has a lot of promise for developing non-invasive glucose monitoring systems [95]. In [96], two spatially separated split-ring resonators are reported, with one interacts with the change in glucose level of a sample under test while the other ring is used as a reference. The comparative analysis of the two split rings following the OGTT protocol is used for validation purposes.

### 6.2. Antenna as Sensors

The change in permittivity with varying glucose concentration can effect both an antenna in near field coupling and electromagnetic transmission. This enables an antenna to work as a sensor following the principles of electromagnetic sensing. To this end, several research work has been reported. In [97], two different antennas: a patch and integrated slot antenna have been designed. The two-port analysis shows the measured gain responds to the glucose concentration change at a frequency of 5.5 GHz using the patch antenna. The slot antenna on the other hand did not produce any glucose sensitive measurements. In [98], the sensor is modeled as a microstrip antenna made of flat sheet of metal separated by a dielectric substrate at a frequency of 2.4 GHz. The return loss for an antenna with microstrip feed is determined to be 36 dB at that frequency. However, the paper does not give any information on the return loss change with varying glucose concentrations. In [99], the sensitivity of the antenna is determined by swarm optimization with a frequency shift of 85.5 MHz corresponding to change in aqueous glucose of 50% and 10%.

In [100], the sensor output is an amplitude-only measurement of the standing wave with frequency on a microstrip line that is spiral shaped with an open-terminated end. A comparative analysis similar to Clarke Error Grid with a commercial device shows promising results. The response of a single-spiral microstrip sensor has been demonstrated to alter significantly in response to changes in recorded blood glucose levels in numerous test participants [101]. A monopole antenna is placed in a cuff and wrapped around the wrist in [102]. Using a realistic tissue model of a human hand, simulations have been performed to obtain the antenna input impedance. The shift in resonant frequency was used to determine the blood glucose level. The frequency targeted for this study is in the range of 1.5–2 GHz. The maximum reflection loss shifts to 1.45 GHz, 1.48 GHz, 1.43 GHz for normoglycemia, hypoglycemia and hyperglycemia, respectively, from 1.78 GHz that is observed in the absence of tissue.

At an operating frequency of 19 GHz, a two-port microstrip line sensor was designed in [103]. A water solution which mimics the permittivity of blood glucose is kept at a tank under the model to fill a slot between the two parts of the substrate. The slot is used for electromagnetic waves to interact with the water glucose solution underneath. The sensor achieves a sensitivity of 2${}^{\circ }$ phase change per 10 mg/dL glucose concentration variation. A truncated microstrip patch antenna was designed in [104]. The sensitivity of the antenna could detect minor changes (10%) in blood’s dielectric characteristics from the transmission coefficient in the range of 21 GHz to 29.6 GHz. In [105], two facing microstrip patch antennas operating at 60 GHz that are placed across the glucose solution are designed. The measured transmission coefficient which depends on the permittivity change along the signal path, was correlated with determine the change in glucose concentration. The work was extended in [106], by utilizing an Intravenous Glucose Tolerance Test for in vivo applications.

**Figure 5:**
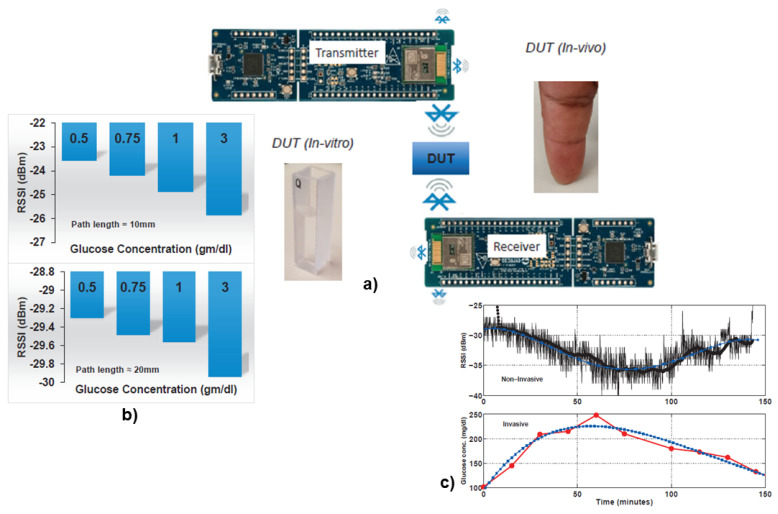
(**a**) Experimental set-up for both the *in-vitro* and *in-vivo* measurements with a bluetooth enabled MCU (Antenna as Sensors) (**b**) The RSSI power level dependence on glucose concentration with *in-vitro* experiment (**c**) Comparison with *in-vivo* experimental results of the noninvasive device with the blood-pricking invasive technique following OGTT protocol on a human subject. Reproduced with permission [107]. Copyright 2019 IEEE.

Chaithanya et al., has implemented the power level of the Received Signal Strength Indicator (RSSI) of a Blutooth Low Energy (BLE) signal at 2.4 GHz to identify the blood glucose level in [107] in a wearable form-factor. Concurrent measurements with the commercial invasive finger pricking approach using the OGTT protocol have shown promising results. This work has been depicted in Figure 5 as an example of sensing with antenna.

The challenges in the detection of blood glucose using electromagnetic method is limited by uncertainty of change in dielectric properties of blood glucose. The sensitivity and selectivity need improvement using advanced machine learning and signal averaging techniques that can mitigate background noise in the data from the contents in the blood to separate high and low amplitude signaling effectively improving the accuracy. Unlike to the previous two optical excitation approaches, this method does not require person specific calibration for skin color differentiation. With the custom development of smart minuscule transceiver system in advanced CMOS technology, the future of this method seems very promising for continuous detection of blood glucose as a non-invasive wearable device (see Figure 5).

## 7. Nanomaterial Based Sensing

**Figure 6:**
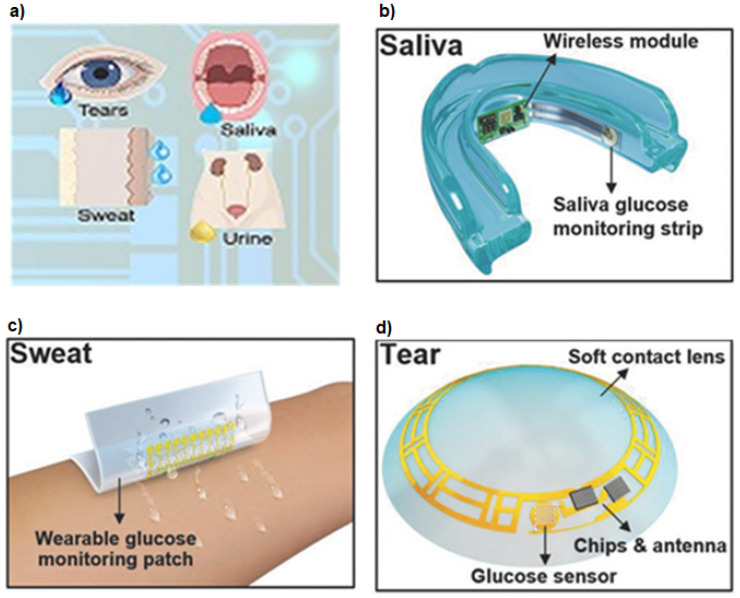
(**a**)The important bio-fluids like tears, saliva, sweat, and urine used to build non-invasive glucose monitoring devices. Typical biosensors based on (**b**) saliva, (**c**) sweat and (**d**) tear. Reproduced with permission [108] Copyright 2022, Ivyspring International Publisher under CC BY. Reproduced with permission [109] Copyright 2018, John Wiley & Sons.

Nanomaterial-based precise glucose monitoring strategies for early-stage detection of diabetes in patients have largely been explored and investigated. Here, we will primarily be discussing nanomaterial-based biosensors which have witnessed extensive applications for precise detection of glucose levels in human urine, saliva, sweat and tears. These techniques are depicted in Figure 6.

Metallic, metal alloy, metal oxide and magnetic nanostructures, carbon-based nanomaterials and even their hybrid/composite structures have been significantly used for non-invasive glucose sensing technologies [110,111,112,113]. Most of these nanomaterials, in particular, metal oxides and ferrite nanoparticles exhibit intrinsic peroxidase-like activity which plays a catalytic role in glucose detection [111,114,115,116,117].

When sugar levels in human blood is on the higher side, excess glucose gets excreted out through urine. The ready availability of urine and its easy collection makes urine-based glucose sensing a non-invasive approach for the diagnosis of diabetes [110]. Su et al. have successfully demonstrated the application of ZnFe${}_{2}$O${}_{4}$ nanoparticles for the detection of glucose levels in urine [111]. The method is based on colorimetric sensing using glucose oxidase (GOx). Recently, colorimetric based sensor using plasmonic gold nanoparticles on graphitic carbon nitride nanosheets (Au nanoparticles @g-C${}_{3}$N${}_{4}$) was used to determine the urine glucose levels [118]. Furthermore, hybrid nanostructures like Co${}_{3}$O${}_{4}$/graphene nanocomposites have been employed as electrode material to detect sugar concentration in urine samples. This is a non-enzymatic electrochemical-based sensor having a detection limit of 0.5 mM [119].

The glucose content in human saliva can also carry useful information regarding the diabetic condition in humans. An early glucose analyzing sensor was developed in [120]. The transducer used here is an enzyme amperometric glucose sensor and has three electrodes. The working electrode made with platinum is covered with an albumin membrane, over which H${}_{2}$O${}_{2}$ based immobilized enzyme membrane is placed. The concentration of H${}_{2}$O${}_{2}$ determines the output current from sensor and which is detected by the amperometry circuit of the saliva analyzing system. A similar study was carried out in [121]. Chakraborty et al. reported fabrication of a non-enzymatic porous CuO nanostructures based electrochemical sensor for salivary glucose monitoring [122]. Similarly, in a recent study, porous NiO nanostructures have been utilized as electrode materials for estimating salivary glucose levels with a detection limit as low as 84 nM [123].

Sometimes morphological changes in nanomaterials have been introduced to offer an enhanced diagnosis approach. Very recently, non-enzymatic and highly sensitive glucose sensors based on composite nanosystems like cuprous oxide nanocubes embedded on graphene have been adopted for a systematic probing of salivary glucose levels [124]. Several other enzyme-free electrochemical-based salivary glucose sensors developed in the recent past based on composite structures include Au nanoparticles on CuO nanorods (∼2009 μA mM${}^{-1}$), IrO${}_{2}$@NiO core–shell nanowires (1439.4 μA mM${}^{-1}$cm${}^{-2}$), CuBr@CuO nanoparticles (3096 μA mM${}^{-1}$cm${}^{-2}$), Cu-Pt nanoparticles on glassy carbon electrode (2209 μA mM${}^{-1}$cm${}^{-2}$), and many more [112,125,126,127].

The physiological data obtained from human sweat or perspiration also possess valuable imprints regarding the glucose concentration in humans. In an attempt to modernize non-invasive glucose detection approaches, several sweat sensors have been developed using nanomaterials of zinc oxide, gold, silver, graphene oxide, carbon nanotubes and their composite systems [128,129,130,131,132,133,134].

Tear based sensing approach using nanomaterials have also gathered significant attention in the development of this kind of non-invasive biosensors [135,136,137,138]. Nanostructures embedded in highly sophisticated and biocompatible contact lenses are capable of detecting glucose levels accurately, sometimes having a detection limit as low as 211 nM [139]. In the commercial arena, a small and flexible spring like device was introduced and termed Noviosense [140].

In the last decade, the nanomaterials have been hugely exploited for advanced biomedical applications [141,142,143,144,145]. As already stated, mostly nano-ferrites and metal oxide nanomaterials as well as graphene oxides and carbon nanotubes show intrinsic peroxidase-like activities [111,116,117,146,147]. Consequently, it is advisable to employ more of these nanostructures, particularly in the form of composites to design and fabricate sophisticated glucose monitoring devices having a much higher degree of sensitivity. Ferrite-based nanostructures are already established candidates in gas sensing applications [148,149]. There lies a good possibility of further exploring these iron oxide-based ferrites for non-invasive glucose sensing applications. Tailoring the physicochemical properties of ferrites and some prominent metal oxide nanostructures, primarily by incorporating suitable dopants (transition metals, rare-earths etc.) and also by introducing shape and surface anisotropies in the system, creating structures like nanowires, nanorods, nano-octopods etc. can possibly have a significant impact in better quantification of glucose levels in humans.

Therefore, these nanomaterial-based sensors have a tremendous future ahead particularly in the design and development of highly sensitive, cost-effective non-invasive glucose monitoring devices. This non-invasive mode of quantifying glucose levels in pre-diabetic and diabetic patients carry immense possibility which could register a giant leap in the healthcare industry as opposed to the traditional blood glucose sensing invasive strategies. These nanomaterial-based biosensors could be the next generation non-invasive technology for efficient and precise quantification of sugar levels in diabetic patients.

## 8. Hybrid, Integrated and Other Methods

Besides these four techniques, some other approaches have also been investigated such as Nuclear Magnetic Resonance [150] at 400 MHz. In [151], a feasibility study of a non-invasive sensor integrating three different types of techniques: electromagnetic, acoustic speed and near infra-red spectroscopy with compensation techniques results. Another study [152] involved an integrated approach combining Near Infra Red (NIR) absorption and bio-impedance measurements using artificial neural network and stochastic methods. An attempt was made to measure the blood glucose level using a capacitance measurement technique in [153] using a parallel plate capacitor, with the forearm in between acting as a dielectric medium. Two metal plates of equal dimensions were placed near left wrist as electrodes. A semi-cylindrical capacitive sensor was similarly proposed in [154]. In [155], the change in dielectric permittivity of saliva estimates the glucose concentration. A correlation between blood glucose levels with saliva glucose was investigated. A microwave biosensor was used at the microwave frequency to measure the shift in resonant frequency of the resonator. However, these techniques or approaches are not widely pursued and investigated so far.

## 9. Conclusions

The aim of this concise and systematic review is to highlight the clinical significance of non-invasive methods in glucose monitoring and bring out the progression of these methods for potential diabetes management in the near future. There are four methods of non-invasive glucose monitoring that were reviewed here and classified primarily based on their principles of detection of the signal from the MUT, which is either a glucose solution (*in-vitro*) or blood glucose (*in-vivo*/*ex-vivo*). These four methods are optical spectroscopy, photoacoustic spectroscopy, electromagnetic sensing and nanomaterial based sensing. Furthermore, integration of these different approaches to bring out a cohesive and hybrid blood monitoring device were also reviewed. The developments observed from all of these investigations suggest that the non-invasive glucose monitoring has a huge potential to replace the current standard of invasive and minimally invasive approaches with a *pain-free*, efficient and accurate diabetes management in the near future. 

## Data Availability

Not applicable.
